# Supplementary figures and images for: Exploring the systemic impacts of urinary tract infection-specific antibiotic treatments on the gut microbiome, metabolome, and intestinal morphology in rats
Source: PeerJ. 2025 Jun 9;13:e19486. doi: 10.7717/peerj.19486 (PMC12161137; doi:10.7717/peerj.19486)

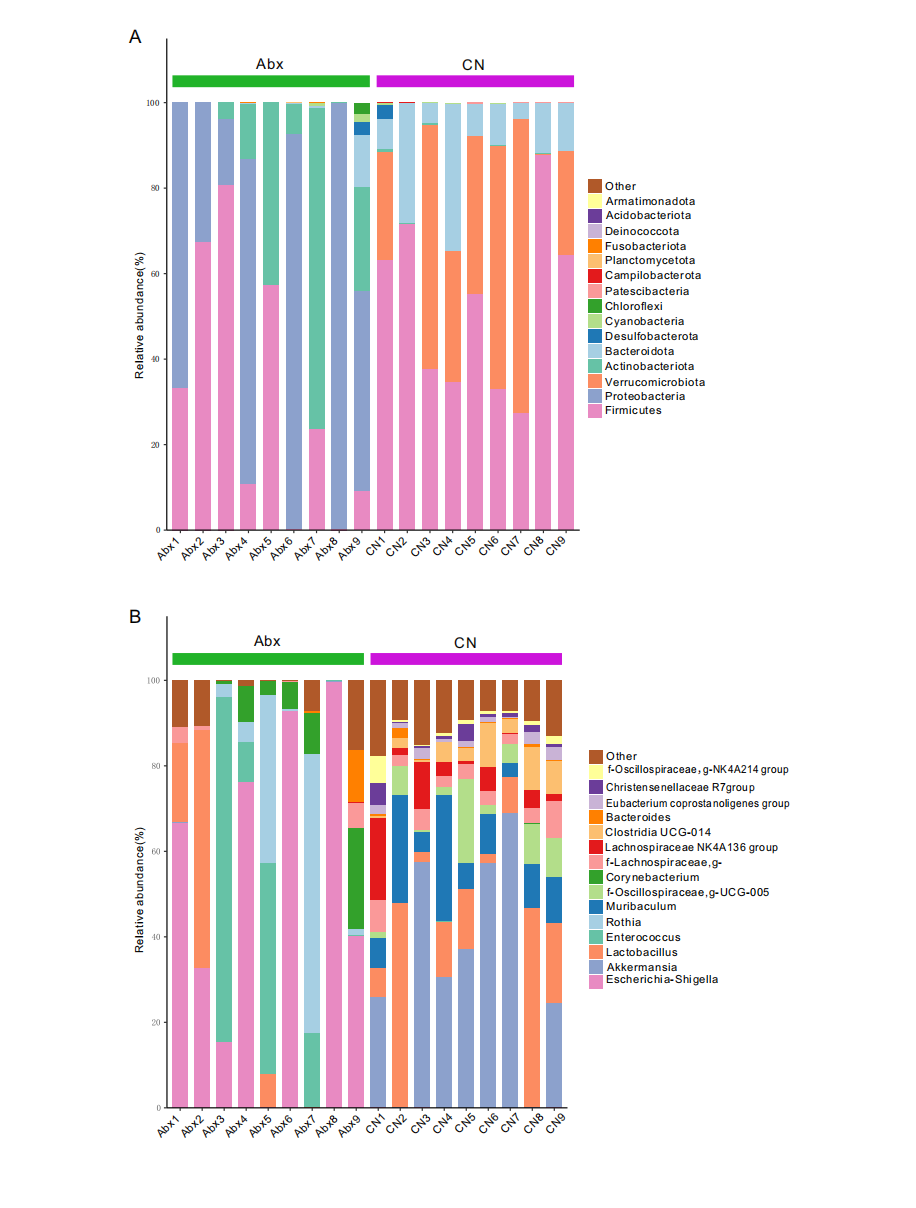

Supplement: Supplemental Information 1 [file peerj-13-19486-s001.png]
