# Supplementary material for: Exploring the systemic impacts of urinary tract infection-specific antibiotic treatments on the gut microbiome, metabolome, and intestinal morphology in rats
Source: PeerJ. 2025 Jun 9;13:e19486. doi: 10.7717/peerj.19486 (PMC12161137; doi:10.7717/peerj.19486)
Supplement: Supplemental Information 2 — Statistical analysis revealed no significant difference between the two groups (p = 0.841). [file peerj-13-19486-s002.docx]

**Table S1** Antimicrobial Peptide Comparison

| **Group** | **mean±SD** | **p-value** |
| --- | --- | --- |
| Abx | 33.13±25.81 | 0.841 |
| Control | 35.30±18.73 |  |

*t*-test was applied.
